# In vitro production of bovine embryos derived from individual donors in the Corral^®^ dish

**DOI:** 10.1186/s13028-017-0309-9

**Published:** 2017-06-15

**Authors:** Maaike Catteeuw, Eline Wydooghe, Erik Mullaart, Hiemke M. Knijn, Ann Van Soom

**Affiliations:** 1 Department of Reproduction, Obstetrics, and Herd Health, Faculty of Veterinary Medicine, Ghent University, Salisburylaan 133, 9820 Merelbeke, Belgium; 2 CRV BV, Wassenaarweg 20, 6843 NW Arnhem, The Netherlands

**Keywords:** Corral^®^ dish, In vitro embryo production, Bovine, Donor

## Abstract

**Background:**

Since the identity of the embryo is of outmost importance during commercial in vitro embryo production, bovine oocytes and embryos have to be cultured strictly per donor. Due to the rather low yield of oocytes collected after ovum pick-up (OPU) per individual cow, oocyte maturation and embryo culture take place in small groups, which is often associated with inferior embryo development. The objective of this study was to improve embryonic development in small donor groups by using the Corral^®^ dish. This commercial dish is designed for human embryo production. It contains two central wells that are divided into quadrants by a semi-permeable wall. In human embryo culture, one embryo is placed per quadrant, allowing individual follow-up while embryos are exposed to a common medium. In our study, small groups of oocytes and subsequently embryos of different bovine donors were placed in the Corral^®^ dish, each donor group in a separate quadrant.

**Results:**

In two experiments, the Corral^®^ dish was evaluated during in vitro maturation (IVM) and/or in vitro culture (IVC) by grouping oocytes and embryos of individual bovine donors per quadrant. At day 7, a significantly higher blastocyst rate was noted in the Corral^®^ dish used during IVM and IVC than when only used during IVM (12.9% ± 2.10 versus 22.8% ± 2.67) (P < 0.05). However, no significant differences in blastocyst yield were observed anymore between treatment groups at day 8 post insemination.

**Conclusions:**

In the present study, the Corral^®^ dish was used for in vitro embryo production (IVP) in cattle; allowing to allocate oocytes and/or embryos per donor. As fresh embryo transfers on day 7 have higher pregnancy outcomes, the Corral^®^ dish offers an added value for commercial OPU/IVP, since a higher blastocyst development at day 7 is obtained when the Corral^®^ dish is used during IVM and IVC.

## Background

Currently, many bovine embryos are being generated in vitro for commercial embryo transfer. In 2013, more than 500,000 embryos have been produced worldwide by ovum pick up (OPU) and in vitro embryo production (IVP) technologies, with South America taking the lead in OPU/IVP. However, during the last decade there has been an almost threefold rise in OPU/IVP embryos produced in Europe and North America, indicating an increasing interest in this application [1]. Since OPU/IVP has become an alternative and highly competitive technique for multiple ovulation and embryo transfer [2], much research has been done to optimize the OPU technique because numerous factors can influence the oocyte yield, such as hormone treatment prior to oocyte collection [3], OPU equipment [4] and interval between OPU sessions [5]. However, an average of only eight oocytes per Holstein–Friesian donor are obtained, a breed particularly used in Europe [6]. When these oocytes are being matured, they are grouped per individual donor, since the genetic identity of the OPU/IVP embryo needs to be preserved. This implies that during OPU/IVP, only small numbers of oocytes and embryos are cultured together. Moreover, quality of oocytes derived after OPU is very variable since some oocytes lack cumulus cells due to vacuum pressure [7]. The quality of the oocyte is however crucial and is predictive of the developmental potential of the resulting embryo [8]. It has been demonstrated that in vitro production starting from oocytes surrounded by few cumulus cells or denuded oocytes resulted in a lower blastocyst formation compared to IVP starting from oocytes surrounded by compact layers of cumulus cells [6, 9]. Due to the scarcity of the oocytes retrieved per donor, a strict selection including only the best cumulus oocyte complexes (COCs) prior to the in vitro process is not always possible. In commercial settings, where oocytes and embryos are cultured separately per donor, an average blastocyst rate of 16–18% is obtained [10, 11]. Besides the low blastocyst yield, there are also indications that grouping small numbers of embryos results in a lower total cell number and a higher rate of apoptosis compared with embryos cultured in large groups (77.16 cells versus 98.48 cells and 24.17% versus 12.14%, respectively) [12]. In mice [13, 14], cattle [15, 16] and humans [17], pooling oocytes and embryos in large groups increases blastocyst yield up to 40%. This beneficial effect of group culture has been related to a higher concentration of embryo secreted factors in the surrounding culture media, such as insulin-like growth factor-I [18] or platelet activating factor [19]. These secreted factors act potentially as a survival factor by preventing apoptosis of the embryonic cells or as a mitogenic factor [20]. In addition, during in vitro maturation cumulus cells and oocytes are also able to secrete signalling molecules. Oocyte secreted factors, such as bone morphogenetic protein 15 and growth differentiation factor 9, regulate a variety of cumulus cell functions associated with growth and differentiation, which in turn may regulate and stimulate the developmental competence of the oocyte [21]. These paracrine and autocrine factors require a close interaction between groups of COCs and subsequently embryos, thus creating a supporting microenvironment for development.

**Fig. 1:**
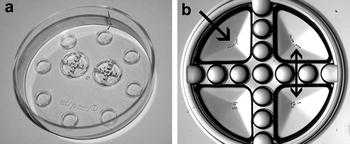
Design of the Corral^®^ dish. **a** The Corral^®^ dish consists of two central wells. **b** These central well is divided into quadrants by a solid wall with numerous posts on top, allowing medium and embryotrophic factors to pass (*double arrow*) but oocytes or embryos remained per individual donor in a quadrant (*arrow*). Each quadrant of the Corral^®^ dish contains the oocytes or embryos of one specific donor and is filled with 30 µL medium

The Corral^®^ dish, designed especially for human in vitro embryo production, consists of two central wells that are divided into four quadrants by a semi-permeable wall (Fig. 1a). In human IVP, a single embryo, having a larger diameter than the gaps between the wall, is placed per quadrant, with a maximum of two times four embryos of the same female patient per Corral^®^ dish. This makes individual monitoring of embryo development possible while the medium and embryotrophic factors can flow through the quadrants (Fig. 1b) [17]. In human studies, single embryo culture has been applied in a Corral^®^ dish setting [17], where only one embryo was placed in one quadrant, but in our study, we chose to allocate eight embryos belonging to the same donor in one quadrant, since embryo culture in small groups is routinely used in bovine OPU/IVP [15]. We hypothesized that the embryonic development would be stimulated in the common medium by the exchange of putative autocrine factors between the donor cows, while individual allocation of oocytes and subsequently embryos per donor cow still remained possible.

## Methods

### Media and reagents

Basic Eagle’s Medium amino acids, Minimal Essential Medium (MEM) non-essential amino acids (100×), TCM-199-medium, kanamycin and gentamycin were purchased from Life Technologies Europe (Ghent, Belgium) and all other components were obtained from Sigma (Schnelldorf, Germany), unless otherwise stated. All the media were filter-sterilized using a 0.22 μm filter (Pall Corporation, Ann Arbor, MI, USA) before use.

### In vitro embryo production protocol

Bovine embryos were produced by adapting previously described routine in vitro methods [22]. Briefly, ovaries were collected per slaughtered cow in separate plastic bags in a local slaughterhouse and processed within 2 h. Follicles between 2 and 8 mm diameter were punctured. Subsequently, COCs and embryos were strictly kept per donor cow during the complete procedure. From each donor, the first 8–10 COCs visible in the Petri dish were collected, without selection based on the quality of these COCs, only denuded oocytes were discarded. If fewer than eight COCs were available, the donor was excluded from the experiments. The COCs were transferred to maturation medium, which consisted of modified bicarbonate-buffered TCM-199 supplemented with 50 µg/mL gentamycin and 20 ng/mL epidermal growth factor (EGF). Subsequently, COCs were matured for 22 h at 38.5 °C in 5% CO_2_ in humidified air.

Fertilization occurred per donor with the semen of the same proven bull. Frozen-thawed spermatozoa were separated over a discontinuous Percoll gradient (45 and 90%; GE Healthcare Biosciences, Uppsala, Sweden). Sperm concentration was adjusted to 1 × 10^6^ spermatozoa/mL using IVF-Tyrode’s Albumin-Pyruvate-Lactate (TALP), which consisted of bicarbonate buffered Tyrode’s solution, supplemented with BSA (Sigma A8806; 6 mg/mL) and heparin (25 μg/mL). The mature oocytes were incubated in 500 µL IVF-TALP with spermatozoa for 21 h at 38.5 °C in 5% CO_2_ in humidified air.

After fertilization, excess spermatozoa and cumulus cells were removed by vortexing. Eight presumptive zygotes per donor were transferred to synthetic oviductal fluid (SOF) supplemented with essential and non-essential amino acids (SOFaa), 0.4% BSA (Sigma A9647) and ITS (5 µg/mL insulin, 5 µg/mL transferrin and 5 ng/mL selenium) and were incubated at 38.5 °C in 5% CO_2_, 5% O_2_ and 90% N_2_ till 8 days post insemination (dpi). During this culture period, embryos were kept in the same culture dish and no renewal of SOF medium was performed.

### Experiment 1: Embryo culture in the Corral^®^ dish

**Fig. 2:**
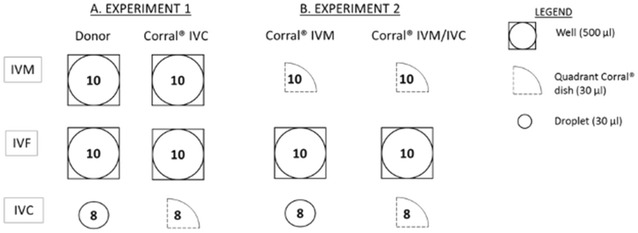
Schematic illustration of the two experiments. As indicated in the legend, oocytes and embryos were grouped per donor in a four-well dish, a droplet or in a quadrant of the Corral^®^ dish during the different phases of the in vitro embryo production (in vitro maturation-IVM, in vitro fertilization-IVF, in vitro culture-IVC). Furthermore, the number of oocytes and embryos grouped together is indicated in the icons.* (A)* In experiment 1, embryos were placed per donor in the Corral^®^ dish or in a separate drop during IVC.* (B)* In experiment 2, oocytes and embryos were assigned per donor to the Corral^®^ dish during IVM or during IVM and IVC

For the first experiment, ovaries from individual donor cows were collected and processed separately. This experiment was conducted 4 times (4 replicates), for each replicate the ovaries of 16 different donor cows were collected. From each donor, 8–10 COCs were matured in 500 µL maturation medium in separate 4-well dishes. Subsequently, the oocytes were fertilized per donor in four-well dishes. After fertilization, the first eight presumptive zygotes were grouped, without prior selection and cultured per donor. Half of these donor groups were allocated to a droplet and the other half to a quadrant of the Corral^®^ dish, this was chosen completely at random. Culture droplets consisted of 30 µL medium, eight droplets were made per culture dish (IVF Petri dish, Nunc^®^, Thermo Fisher, Denmark) and 8.5 mL mineral oil was covering these droplets (Drop IVC). In the Corral^®^ dish, the two central wells were filled with 120 µL culture medium, each quadrant containing 30 µL (Corral^®^ IVC). Mineral oil (8.5 mL) was used to cover the droplets of medium to prevent evaporation. Because of the typical structure of the Corral^®^ dish, zygotes of eight different donors were grouped in those two central wells. An overview of COC and embryo distribution is shown in Fig. 2a.

### Experiment 2: Oocyte maturation and embryo culture in the Corral^®^ dish

Comparable to experiment 1, this experiment was conducted four times (4 replicates). For each replicate, ovaries from 16 different donors were collected separately. From each donor, ten COCs were matured in a quadrant of the Corral^®^ dish. Each quadrant contains 30 µL maturation medium. In one central well, 40 COCs of four different donors were matured in 120 µL medium, each donor separated by the semi-permeable wall dividing the Corral^®^ dish in quadrants. Both central wells were covered with 8.5 mL mineral oil. Routine fertilization occurred per donor in four-well dishes. As described in the first experiment, eight presumptive zygotes were cultured per donor either in a 30 µL drop of medium (Corral^®^ IVM) or in a quadrant of the Corral^®^ dish (Corral^®^ IVM/IVC). In the latter, eight different donors were again grouped in the two central wells. An overview of COC and embryo distribution is shown in Fig. 2b.

### Evaluation of embryo development and embryo quality

To evaluate the embryo development, the cleavage rate was assessed at 45 h post insemination (hpi) as the percentage of presumed zygotes that cleaved. Blastocyst stages were evaluated according to the fourth edition IETS manual at 7 and 8 dpi. At 8 dpi, hatching rate was evaluated as the percentage of hatching or hatched blastocysts. Subsequently, total cell number (TCN) of the blastocysts was assessed by Hoechst 33342 staining. Briefly, day 8 blastocysts were fixed in 2% paraformaldehyde during 20 min and subsequently stained for 10 min with 0.1% Hoechst 33342. The stained blastocysts were evaluated using a 400× magnification fluorescence microscope (Leica DM 5500 B).

### Statistical analysis

Statistical analyses were carried out with IBM SPSS Statistics 23. Differences at *P*-value <0.05 were considered statistically significant. Cleavage, blastocyst and hatching rates were analysed using a binary logistic regression model with treatment (Drop IVC vs Corral^®^ IVC and Corral^®^ IVM vs Corral^®^ IVM/IVC) and replicate as fixed effects. The effect of replicates was assessed and excluded from the final model if it was not significant. Total cell numbers were analysed using a mixed model analysis of variance, with treatment (Drop IVC vs Corral^®^ IVC and Corral^®^ IVM vs Corral^®^ IVM/IVC) as fixed effect and replicate as random effect and are expressed as mean ± standard error of the mean (SEM). If the effect of replicates was not significant, this was excluded from the final model.

## Results

### Experiment 1: Embryo culture in the Corral^®^ dish

There was no significant difference noted in embryonic development between embryos cultured in individual donor droplets (Drop IVC) or embryos cultured in the Corral^®^ dish (Corral^®^ IVC) (Table 1). Both cleavage and blastocyst rate were similar in both groups. At day 8, a blastocyst rate was reached of 26.9% in Corral^®^ IVC and 24.8% in Drop IVC. Furthermore, no differences were observed in TCN of day 8 blastocysts (Drop IVC: 188.1 ± 10.49; Corral^®^ IVC: 194.2 ± 13.59).

**Table 1:** Embryonic development in the different treatment groups of experiments 1 and 2

	Treatment	Number of oocytes	Cleavage (%)	Blastocysts D7 (%)	Blastocysts D8 (%)	% Hatched
Exp 1	Drop IVC	219	158 (72.1)	41 (18.7)	59 (26.9)	22.0
	Corral^®^ IVC	234	169 (72.2)	38 (16.2)	58 (24.8)	17.2
Exp 2	Corral^®^ IVM	255	186 (72.9)	33 (12.9)*	68 (26.7)	22.1
	Corral^®^ IVM/IVC	246	191 (77.6)	56 (22.8)*	74 (30.1)	28.4

### Experiment 2: Oocyte maturation and embryo culture in the Corral^®^ dish

Cleavage rate did not differ between two groups. Significantly more blastocysts were observed in the Corral^®^ IVM/IVC compared to the Corral^®^ IVM, at 7 dpi (P < 0.05), respectively 22.8 and 12.9%. This was however no longer the case for blastocyst rate at day 8, 26.7% was reached in the Corral^®^ IVM and 30.1% in Corral^®^ IVM/IVC (Table 1). Furthermore, TCN of day 8 blastocysts in these two groups did not differ (Corral^®^ IVM: 125.0 ± 3.21; Corral^®^ IVM/IVC: 133.9 ± 3.87).

Cleavage (45 hpi), blastocyst (7 and 8 dpi) and hatching rates of embryos produced per donor in droplets (Drop IVC) or in a quadrant of the Corral^®^ dish (Corral^®^ IVC) during in vitro culture (experiment 1) and embryos produced per donor in a quadrant of the Corral^®^ dish during in vitro maturation (Corral^®^ IVM) or during in vitro maturation and culture (Corral^®^ IVM/IVC) (experiment 2). Asterisks (*) in the same column indicate a statistical difference between treatments within the same experiment (P < 0.05).

## Discussion

The overall aim of this study was to evaluate the efficiency of Corral^®^ dish for commercial purposes. The Corral^®^ dish allows grouping of embryos from different donor groups without losing track of genetic identity by placing the embryos of each donor in a quadrant of the central wells. This allows secreted embryotrophic factors to reach a larger group of embryos (32 instead of 8) since all embryos are exposed to the same surrounding medium. These factors stimulate growth and development, which would result in a higher blastocyst yield. In this study, there was no difference found in embryonic development when applying the Corral^®^ dish during culture in comparison with the allocation of embryos per donor in a separate droplet of medium. This was similar to the study of Ebner et al. [17] on human embryos, where one embryo was allocated to either a quadrant of the central wells or one embryo allocated to one of the other wells. However, when the Corral^®^ dish was used both during maturation and culture, blastocyst yield was increased at 7 dpi compared to its use only during maturation, but this effect was no longer noticed at 8 dpi. Because more embryos reach the blastocyst stage on day 7 in the Corral^®^ dish, when used during IVM and IVC, it offers the opportunity to transfer more fresh IVP embryos, which may subsequently give rise to more pregnancies and live born calves. It has been reported that pregnancy outcome is the highest when transferring fresh day 7 in vitro blastocysts, after transfers with in vivo derived embryos [23, 24].

**Fig. 3:**
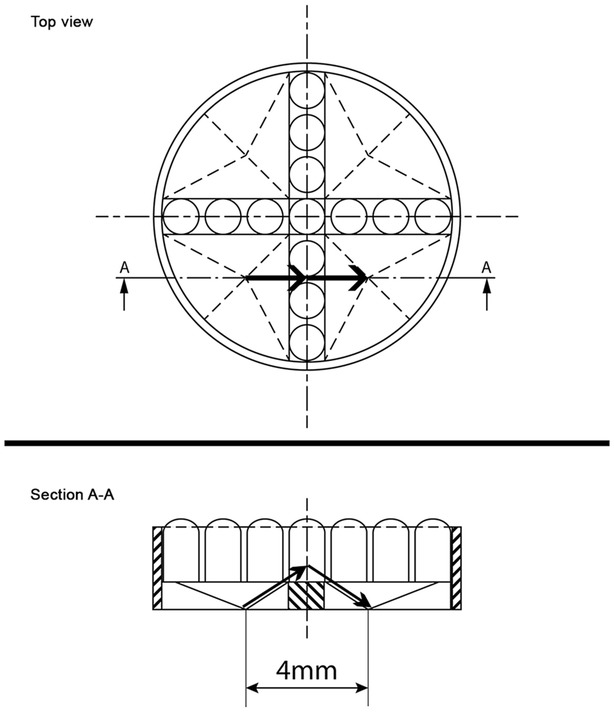
Graphic design of the Corral^®^ dish. This figure is pointing out a distance of 4 mm between the deepest sites of the quadrants. An even larger distance has to be covered by the embryotrophic factors, secreted by the allocated cumulus–oocyte complexes or embryos, to reach another quadrant. The diffusion of secreted factors can only appear in a vertical and oblique direction (*bold arrows*), due to the well-shaped quadrants and the in-between wall

On the other hand, the Corral^®^ dish, has a specific design which implicates also three main disadvantages. First, the distance between donor groups is over 4 mm (Fig. 3). Gopichandran and Leese [16] reported that an optimal blastocyst formation occurred when a distance of 165 µm between the embryos was achieved. In the Corral^®^ dish, the distance components in the medium have to cross between two donor groups is probably too large for optimal exchange of autocrine factors. A mathematical model constructed by Matsuura [25] calculated the concentration of secreted factors by embryos cultured in microwells and concluded that macromolecules (growth factors) are slowly diluted and remain quite high in the neighbourhood of the embryo; while small molecules (waste materials) are rapidly diffusing away from the embryo. Due to the sloped sides of the quadrants (Fig. 3), diffusion of secreted factors could therefore be facilitated in a vertical and oblique direction, with growth factors remaining in the neighbourhood of the embryos located in the deepest point of the well. Second, each quadrant needs to be filled with 30 µL medium in order to connect the four quadrants. In this way, adjusting the incubation volume to the number of oocytes or embryos is impossible. This static design has therefore a major impact on oocyte/embryo density, which is referring to the number of embryos on a given amount in micro liter of medium and which is an important parameter during IVP [26–30]. Because of low embryo numbers in commercial practices, the medium volume cannot be decreased to achieve the ideal density of 1:1–1:3 [31–33]. The design of the Corral^®^ dish could therefore be more suitable for donor cows having large numbers of COCs, since this is the only way to acquire a high embryo density in the Corral^®^ dish. In Nelore cattle, a typical Brazilian breed, 30 or more oocytes can be collected per OPU session and this breed is therefore an excellent candidate for providing embryos for culture in the Corral dish [34]. Finally, from a sanitary point of view, possible transmission of pathogens can be considered as a risk factor between oocytes and embryos of different donors grouped in the Corral^®^ dish. In theory, infection can be present as a consequence of intrafollicular infection or in vitro fertilization with infected semen. However, the risk of infection is rather small, since donor cows are carefully selected and tested for the absence of specific viral infections like bovine herpesvirus type 1 before entering an in vitro embryo program, and also every bull is tested for absence of infectious pathogens before he is allowed to enter an artificial insemination program. The zona pellucida plays a major role in protecting the embryo, and only very small viruses can form a risk for transzonal infection. Furthermore, before embryo transfer, washing embryos in combination with trypsin treatment is advised by sanitary procedures of the International Embryo Technology Society [35] to inactivate and remove possible viruses. In the end, it should still be advised to group oocytes and embryos of different donor cows in the Corral^®^ dish only when the full health status is known.

## Conclusions

A novel aspect of this study was that we used the Corral^®^ dish for grouping small numbers of bovine oocytes and embryos per donor cow, whereas in other studies embryos have been cultured singly, thereby decreasing a possible beneficial effect of group culture. It is however doubtful whether the embryos can benefit from being grouped in the Corral^®^ dish. Nonetheless, the Corral^®^ dish is an easy applicable tool to produce in vitro embryos by grouping bovine oocytes and embryos per donor. Furthermore, the Corral dish^®^ increases blastocyst development at 7 dpi, when used during IVM and IVC, and is therefore beneficial for commercial practice regarding embryo transfers as higher pregnancy rates are achieved with fresh day 7 blastocysts.

## Abbreviations

COCs: cumulus oocyte complexes
dpi: days post insemination
IVC: in vitro culture
IVM: in vitro maturation
IVP: in vitro production
OPU: ovum pick up
TCN: total cell number
